# Hepatitis B status and associated factors among participants screened for simulated HIV vaccine efficacy trials in Kenya and Uganda

**DOI:** 10.1371/journal.pone.0288604

**Published:** 2023-07-17

**Authors:** Yunia Mayanja, Wasima Rida, Joshua Kimani, Ali Ssetala, Juliet Mpendo, Annet Nanvubya, Gaudensia Mutua, Omu Anzala, Matt A. Price

**Affiliations:** 1 Medical Research Council/ Uganda Virus Research Institute and London School of Hygiene and Tropical Medicine (MRC/UVRI & LSHTM) Uganda Research Unit, Entebbe, Uganda; 2 Biostatistics Consultant, Arlington, Virginia, United States of America; 3 SWOP-PHDA, University of Nairobi/University of Manitoba, Nairobi, Kenya; 4 Uganda Virus Research Institute/ International AIDS Vaccine Initiative (UVRI-IAVI) HIV Vaccine Programme, Entebbe, Uganda; 5 IAVI, Nairobi, Kenya; 6 KAVI- Institute for Clinical Research, Nairobi, Kenya; 7 Department of Epidemiology and Biostatistics, University of California San Francisco, San Francisco, CA, United States of America; 8 IAVI, New York, New York, United States of America; Uniformed Services University: Uniformed Services University of the Health Sciences, UNITED STATES

## Abstract

**Introduction:**

Hepatitis B (HBV) prevalence remains high in Sub Saharan Africa and among some key populations such as those with continued exposure through sexual contact. We assessed the HBV status among potential participants who were screened for simulated HIV vaccine efficacy trials in Kenya and Uganda.

**Methods:**

We conducted a cross sectional analysis of data collected from individuals who were screened in Kenya (Nairobi) and Uganda (Entebbe and Kampala). The studies followed hypothetical procedures of an HIV vaccine efficacy trial and aimed to enroll HIV negative key and vulnerable populations at elevated risk of HIV acquisition. HBV status was the main outcome categorized using Hepatitis B surface antigen (HBsAg) and total Hepatitis B core antibody (HBcAb). Baseline characteristics potentially associated with never being infected were analyzed using logistic regression.

**Results:**

We screened 1,366 participants with mean age (SD) 28.7 (7.3) years. Overall, 46.6% were from Entebbe, 50.7% had secondary or higher level of education, 76.4% had informal high-risk jobs and 56.3% were male. Kampala had only female participants contributing 60.6% of females screened. Of the screened participants, 94.7% and 3.4% were negative and positive for HBsAg respectively. The prevalence on HBV infection was 3.9% among males and 2.8% among females while prevalence by site was: Entebbe (4.9%); Kampala (4.1%) and Nairobi (0.3%). The highest HBV prevalence was found among participants aged 25-29-years (5.2%), those with primary level education (4.5%), and those in informal low risk jobs (6.5%). Considering 1265 participants with complete data on HBsAg and HBcAb-Total, HBV status was never infected (67.9%), past infection (28.5%), chronic infection (3.2%) and acute infection (0.5%). Of 859 who were never infected, 685 (79.7%) were tested for anti-HBs titers of whom 60 (8.8%) had titers >10IU/L (immune due to vaccination). The odds of never being HBV infected were lower among older individuals 25–29 years (AOR 0.51; 95%CI 0.36–0.71) and ≥30 years (AOR 0.35; 95% CI 0.25–0.49). The odds were higher among participants with informal high-risk jobs from Kampala (AOR 2.21; 95% CI 1.41–3.47) and Nairobi (AOR 2.61; 95% CI 1.72–4.00) compared to those from Entebbe.

**Conclusion:**

HBV prevalence and immunity due to vaccination were low among HIV negative individuals who are eligible for HIV vaccine trials and prevalence varies by age, education level and main occupation. Younger individuals and those recruited from existing cohorts/ clinics have a higher likelihood of having no prior HBV infection. HIV prevention intervention trials are a platform to identify individuals that need HBV vaccination.

## Introduction

Worldwide in 2019, an estimated 1.5 million people acquired hepatitis B virus (HBV), a chronic infection of public health concern because it is complicated by liver cirrhosis and primary liver cancer in 20–30% of individuals [1]. Globally, there were 316 million people living with chronic HBV in 2019 giving a prevalence of 4.1%. Of these, 555,000 people died from Hepatitis related causes [2] surpassing HIV related deaths by 140,000 despite both infections having similar infection rates that year [3]. The Sub Saharan Africa (SSA) region had the second highest HBV prevalence globally in 2019 (6.5%) and accounted for 71,000 (13%) of HBV related deaths in 2019 [2]. Surveys done among the general population in different parts of SSA show that the prevalence of HBV infection ranges from 6.0–17.6% [4–7]. Whereas HBV prevalence has been widely documented among the general population in SSA, similar data among different key populations (KPs) living with and without HIV are still limited. Indeed, KPs in SSA are at risk of acquiring other sexually transmitted infections such as syphilis, chlamydia and gonorrhea [8, 9] and blood borne infections such as Hepatitis C [10] which have similar modes of transmission to HIV and HBV.

Recent surveys done among KPs living with HIV show varied HBV prevalence, including 4.0% from a cross-sectional survey among female sex workers (FSWs), men who have sex with men (MSM) and injection drug users (IDUs) in South Africa (prevalence similar across KPs) [11] and 32.8% among IDUs in Mozambique [12]. A study done in Nigeria indicates 17.1% HBV prevalence among FSWs however data was not disaggregated by HIV status and yet HIV/HBV co-infection causes mutual detrimental effect on disease progression. Studies done among people living with HIV show that HIV infection accelerates HBV-related liver damage, leading to earlier cirrhosis and end-stage liver disease [13] and similarly, HBV infection accelerates HIV progression [14], impairs immunological recovery [15] and increases HIV related morbidity and mortality [13]. Among HIV negative KPs and other populations considered eligible for HIV prevention intervention trials in some settings, e.g., fisher folk, understanding the HBV disease burden is also important. Firstly, HBV infection may serve as trial exclusion criteria as in the HIV Prevention Trials Network (HPTN083) trial that compared efficacy of long acting injectable cabotegravir to daily oral Tenofovir Disoproxil Fumarate/Emtricitabine (TDF/FTC) for HIV pre-exposure prophylaxis (PrEP) [16]. Secondly, the components of oral PrEP (TDF/ FTC) are also active against HBV infection. Given the current HIV vaccine trial designs which allow access to or actively provide oral PrEP [17] a few individuals living with HBV who have sub-optimal PrEP use during the trial may have flare ups of HBV infection [18, 19]. Clinical guidelines for PrEP therefore recommend screening tests for Hepatitis B surface Antigen (HBsAg). Furthermore, it is important to identify individuals who are living with chronic HBV infection as a proportion of them will be eligible for life-long HBV treatment [20].

Simulated HIV vaccine efficacy trials (SiVETs) using licensed Hepatitis B vaccine (ENGERIX-B™ GlaxoSmithKline Biologicals Rixensart, Belgium and EUVAX by SANOFI Pasteur) to approximate the vaccination schedules and conditions of an HIV vaccine trial have been conducted among KPs in SSA. The SiVETs reported on vaccination completion and retention [21], estimating HIV incidence for actual HIV vaccine trials [22] and willingness of KPs to participate in future HIV vaccine trials [23, 24]. However, data remains limited on HBV prevalence among potential HIV vaccine trial participants. We conducted a cross sectional study to assess the HBV status among participants who presented for study screening in the SiVET studies.

## Materials and methods

### Study design

We conducted a cross sectional analysis of data collected from individuals who were screened for SiVET studies at three sites: two in Uganda and one in Kenya.

### Study populations

All three SiVETs, following the hypothetical procedures of an HIV vaccine efficacy trial, aimed to enroll key and vulnerable populations at elevated risk of HIV acquisition. Each site followed a similar visit schedule and study procedures but focused on different populations based on site experience and expertise. The Uganda sites in Entebbe and Kampala enrolled fisher folk and FSWs respectively while the Kenya site in Nairobi enrolled FSWs and MSM. The study teams worked closely with community leadership, peer leaders, and existing Community Advisory Boards (CABs) to identify and educate potential participants and ensure that recruitment procedures were acceptable. Study information was given through community information seminars, on-site participant meetings and during the CAB meetings. The Kampala and Nairobi study teams enrolled from pre-existing cohorts and the Entebbe study team enrolled from fishing communities. Interested participants were told about eligibility criteria (e.g., at risk of HIV acquisition) and invited for study screening.

### Study settings and sampling

#### Entebbe site

The Uganda Virus Research Institute/ International AIDS Vaccine Initiative (UVRI-IAVI) HIV vaccine program conducted the study in two conveniently selected fishing communities (FCs) on Lake Victoria, one island and one on the mainland. Volunteers were randomly selected from the two FCs. Screening was conducted from December 2015 to July 2017 until a cohort of 250 SiVET participants (80 from the island, 170 from the mainland) was enrolled.

#### Kampala site

The study was conducted at the Good Health for Women Project (GHWP) clinic of the Medical Research Council/ Uganda Virus Research Institute and London School of Hygiene and Tropical Medicine (MRC/UVRI and LSHTM) Uganda Research Unit. The clinic was in a peri-urban community in southern Kampala and enrolled and followed up a cohort of FSWs including emancipated/ mature minors according to national guidelines [25]. The GHWP cohort screened participants for HIV every 3 months; this process effectively pre-screened for those living with HIV who were therefore not referred for SiVET screening procedures. Screening of women attending the GHWP clinic was conducted from August 2014 to April 2016 and women were consecutively enrolled until a cohort of 290 SiVET volunteers was achieved. The Kampala site enrolled only females.

#### Nairobi site

The KAVI- Institute for Clinical Research (KAVI-ICR), conducted the study at the college of health sciences, University of Nairobi. KAVI-ICR set enrollment numbers at 80% MSM, 20% FSWs. Screening was conducted from the Sex Workers Outreach Program (SWOP-Kenya) clinics in Nairobi from September 2015 to September 2017 and volunteers referred to KAVI-ICR until a cohort of 250 SiVET volunteers was enrolled.

### Eligibility and screening procedures

Potential volunteers were invited for SiVET study screening if they had been community residents in the FCs for at least 6 months (Entebbe), had been attending the GHWP clinic for 6 to 18 months and had tested HIV negative at the last clinic visit (Kampala) or had been enrolled for at least 6 months in the SWOP network of clinics in Nairobi (Nairobi). As part of the screening process for all the SiVET studies, volunteers gave a blood sample to test for Hepatitis B infection and HIV, and a urine sample for pregnancy. Volunteers were not eligible for the SiVET if they were found to be pregnant or living with HIV.

### Laboratory procedures

HIV testing was performed on serum using two or more rapid antibody diagnostic tests administered serially (Entebbe and Kampala) as follows: Determine screening test (Alere Determine^TM^ HIV-1/2Alere Medical Co., Ltd, Japan) Statpak rapid confirmatory kit (HIV-1/2 Statpak Dipstick, CHEMBIO Diagnostic systems, INC. USA) and Unigold as tie breaker (UnigoldTM HIV, Trinity Biotech PLC. Ireland). In Nairobi, rapid antibody tests were administered in parallel as follows: Determine screening test (Alere Determine^TM^ HIV-1/2Alere Medical Co., Ltd, Japan) and Unigold (UnigoldTM HIV, Trinity Biotech PLC. Ireland) followed by BIOELISA as tie breaker. Pregnancy tests were performed on urine using QuickVue one step hCG Combo test strips (Quidel Corporation, San Diego CA, USA).

All sites measured hepatitis B surface antigen (HBsAg) and total hepatitis B core antibody (HBcAb (Total)). The site in Kampala also measured IgM antibodies to core protein (HBcAb (IgM)) while all sites measured antibodies to surface protein (anti-HBs) in at least a subset of volunteers who were also screened for an immunology sub-study. This sub-study aimed to assess how the pre-existing immune status affected responses to Hepatitis B vaccination and therefore needed individuals who had never been exposed to Hepatitis B either through vaccination or prior infection. The assays used to test serum samples for Hepatitis B at the 3 sites are shown below (Table 1).

**Table 1:** Assays used by participating research centers to screen for Hepatitis B.

Test	Entebbe	Kampala	Nairobi
Hepatitis B Surface Antigen (HBsAg)	VIDAS HBsAg Ultra	Roche Cobas e 411	Bioelisa HBsAg V3.0
Hepatitis B Core Antibodies (HBcAb (Total)	VIDAS Anti-HBc Total 11 (HBCT)	Roche Cobas e 411	Bioelisa Anti-HBc
Hepatitis B Core Antibodies (HBcAb (IgM)	Not done	Roche Cobas e 411	Not done
Anti-Hepatitis B Antibodies (Anti-HBs)	VIDAS Anti-HBs Total II (AHBS)	Roche Cobas e 411 (Done for a sub-set who were HBV naïve at screening	Bioelisa Anti-HBs (Done for a sub-set who were HBV naïve at screening)

### Study outcome variables

‘HBV status’ was the main outcome. We used HBsAg and HBcAb (Total) to categorize participants’ HBV status as follows:

- *Living with HBV* if HBsAg was positive. Test results that were HBsAg positive, but HBcAb (Total) negative were further classified as *acute infection* while those that were positive on both tests were classified as *early or chronic infection*.
- *No prior HBV infection* if negative on both tests.
- *Having Past infection* if HBsAg was negative and HBcAb (Total) positive.
- *Unknown HBV Status* if HBsAg was missing.

For the subset of participants with anti-HBs results, participants who were considered as having no prior HBV infection were further categorized as immune due to vaccination if anti-HBs titers were >10IU/L. Otherwise, the participants were defined as non-immune.

Independent variables: While sites varied in the baseline data they collected, common variables included age, sex, education, occupation, and religion. HIV status was not included as referral to SiVET was limited to HIV negative individuals (and participating sites had different pre-screening efforts to reduce the likelihood of identifying persons with HIV at the screening visit). Given the broad range of activities of the participants, main occupation was categorized into three as follows:

- *Formal employment* e.g., teacher, government employee, health worker, military worker, factory worker.
- *Informal high risk* e.g., fisherman, bar or lodge attendant/ owner, saloon attendant, sex worker, truck/taxi driver, bike/boat rider, working in a massage parlor, unemployed.
- *Informal low risk* e.g., farmer, shopkeeper, market vendor, household worker, mason.

### Statistical analysis

Data analyses were conducted using STATA version 16.0 (Stata Corporation, College Station, Texas, USA) and R version 4.2.1 (http://CRAN.R-project.org). The binary outcome of “no prior HBV infection” was used in a univariable and multivariable logistic regression analysis to assess its association with baseline variables. The analysis included only those participants who had complete data on HBsAg and HBcAb (Total). Participants who were missing data for a given baseline variable were excluded from the analysis of that variable. No missing data were imputed. Given that occupation was confounded by site, we created a 6-level categorical variable for site by occupation. Variables for which the unadjusted association attained statistical significance at the p≤0.05 level using a likelihood ratio test (LRT) were considered for the multivariable model. Finally, the association of the outcome with independent variables was assessed and considered statistically significant at p<0.05. Results are presented as adjusted odds ratios (AOR) and 95% confidence intervals. A secondary analysis with the binary outcome of “living with HBV” was performed to offer further insights.

### Ethical considerations

The Uganda National Council for Science and Technology (HS 1850, HS 1584) and Uganda Virus Research Institute-Research Ethics Committee (GC/127/15/07/439, GC/127/14/04/454) approved the Entebbe and Kampala SiVET studies, respectively. The Nairobi SiVET was approved by Kenyatta National Hospital/University of Nairobi Ethics and Research Committee (P137/03/2015). We obtained written informed consent from all participants before any study procedures.

## Results

### Socio-demographic characteristics of participants screened for SiVET studies in Kenya and Uganda (2014–2017)

As shown in Table 2 below, the studies screened 1,366 participants of whom 769 (56.3%) were males; The mean age (SD) was 28.7 (7.3) years with range 16–57 years, 46.6% were from Entebbe, 36.7% were ≥30 years, and 50.7% had secondary level education or higher. Of 769 male participants, 62.4% were from Entebbe. Kampala had only female participants contributing 60.6% (362/597) of females screened.

**Table 2:** Socio-demographic characteristics by site of participants screened for SiVET studies in Kenya and Uganda (2014–2017).

		Total		Entebbe		Kampala		Nairobi	
Screening Dates				Oct2015—Jun2016		Aug2016—May2016		Oct2015—Sep2017	
Characteristic	Categories	N	col %	N	row %	N	row %	N	row %
Total		**1366**	**100.0**	636	46.6	362	26.5	368	26.9
Sex									
	Female	597	43.7	156	26.1	362	60.6	79	13.2
	Male	769	56.3	480	62.4	0	0.0	289	37.6
Age at screening (years)									
	16–24 years	441	32.3	185	42.0	106	24.0	150	34.0
	25–29 years	424	31.0	201	47.4	135	31.8	88	20.8
	30–57 years	501	36.7	250	49.9	121	24.2	130	25.9
Education									
	None	50	3.7	25	50.0	21	42.0	4	8.0
	Primary	506	37.0	266	52.6	196	38.7	44	8.7
	Secondary+	692	50.7	345	49.9	145	21.0	202	29.2
	Missing	118	8.6	0	0.0	0	0.0	118	100.0
Main Occupation									
	Formal	108	7.9	108	100.0	0	0.0	0	0.0
	Informal High Risk	1043	76.4	319	30.6	356	34.1	368	35.3
	Informal Low Risk	215	15.7	209	97.2	6	1.4	0	0.0
Religion									
	Christian	1115	81.6	532	47.7	273	24.5	310	27.8
	Muslim	202	14.8	96	47.5	89	44.1	17	8.4
	Other	48	3.5	8	16.7	0	0.0	40	83.3
	Missing	1	0.1	0	0.0	0	0.0	1	100.0

Entebbe had the biggest proportion of participants aged ≥30 years (49.9%, n = 501) and those with secondary education or higher (49.9%, n = 692). Informal high-risk jobs were reported by 76.4% of participants and Entebbe was the only site with participants having formal jobs (7.9%) as their main occupation. Details in Table 2.

### Hepatitis B status of participants screened for SVET studies in Kenya and Uganda by baseline characteristics (2014–2017)

Table 3 presents the HBsAg results (i.e., test for current HBV infection) by site and socio-demographic characteristics of 1,366 screened participants. Overall, 94.7% were negative for HBsAg, 3.4% were positive and 1.9% of participants did not have laboratory results (unknown HBV status). Considering HBV status of 1,265 participants with complete data on HBsAg and HBcAb-Total, 67.9% had no prior HBV infection, 28.5% had past infection, 3.2% had chronic infection and the rest had acute infection. Of 859 who had no prior HBV infection, 685 (79.7%) were tested for anti-HBs titers of whom 60 (8.8%) had titers >10IU/L (suggesting immunity due to vaccination).

**Table 3:** Hepatitis B status of participants screened for the SiVET studies in Kenya and Uganda by baseline characteristics.

			HBsAg Result						HBV Status (Primary Analysis Set)								
			Positive		Negative		Unknown			No prior HBV Infection		Past Infection		Acute Infection		Early/Chronic Infection	
Characteristic	Category	N	n	%	n	%	n	%	N	n	%	n	%	n	%	n	%
1366		**1366**	47	3.4	1293	94.7	26	1.9	**1265**	859	67.9	360	28.5	6	0.5	40	3.2
Site																	
	Entebbe	636	31	4.9	602	94.7	3	0.5	558	318	57.0	210	37.6	1	0.2	29	5.2
	Kampala	362	15	4.1	344	95.0	3	0.8	359	267	74.4	77	21.4	5	1.4	10	2.8
	Nairobi	368	1	0.3	347	94.3	20	5.4	348	274	78.7	73	21	0	0	1	0.3
Sex																	
	Female	597	17	2.8	572	95.8	8	1.3	577	406	70.4	154	26.7	5	0.9	12	2.1
	Male	769	30	3.9	721	93.8	18	2.3	688	453	65.8	206	29.9	1	0.1	28	4.1
Age Group																	
	<25	441	8	1.8	428	97.1	5	1.1	403	326	80.9	70	17.4	2	0.5	5	1.2
	25–29	424	22	5.2	396	93.4	6	1.4	402	268	66.7	112	27.9	2	0.5	20	5.0
	≥30	501	17	3.4	469	93.6	15	3.0	460	265	57.6	178	38.7	2	0.4	15	3.3
Education																	
	None	50	2	4.0	47	94.0	1	2.0	48	24	50	22	45.8	0	0.0	2	4.2
	Primary	506	23	4.5	482	95.3	1	0.2	465	295	63.4	148	31.8	6	1.3	16	3.4
	Secondary+	692	21	3.0	667	96.4	4	0.6	654	463	70.8	170	26	0	0.0	21	3.2
	Missing	118	1	0.8	97	82.2	20	16.9	98	77	78.6	20	20.4	0	0.0	1	1.0
Main Occupation*																	
	Formal	108	3	2.8	104	96.3	1	0.9	98	50	51.0	45	45.9	0	0.0	3	3.1
	Informal HR	1043	30	2.9	990	94.9	23	2.2	972	683	70.3	260	26.7	5	0.5	24	2.5
	Informal LR	215	14	6.5	199	92.6	2	0.9	195	126	64.6	55	28.2	1	0.5	13	6.7
Religion																	
	Christian	1115	40	3.6	1052	94.3	23	2.1	1034	694	67.1	301	29.1	5	0.5	34	3.3
	Muslim	202	7	3.5	192	95.0	3	1.5	183	130	71	46	25.1	1	0.5	6	3.3
	Other	48	0	0.0	48	100	0	0.0	47	34	72.3	13	27.7	0	0.0	0	0.0
	Missing	1	0	0.0	1	100	0	0.0	1	1	100	0	0.0	0	0.0	0	0.0

*HR = High Risk; LR = Low Risk

The prevalence of HBV infection was 3.9% among males and 2.8% among females while prevalence by site was: Entebbe (4.9%); Kampala (4.1%) and Nairobi (0.3%). The highest HBV prevalence was found among participants aged 25-29-years (5.2%), those with primary level education (4.5%), and those in informal low risk employment (6.5%). The proportion with chronic infection was higher than that with acute infection in all age groups i.e., <25 years (1.2% vs 0.5%); 25–29 years (5.0% vs 0.5%) and ≥30 years (3.3% vs 0.4%) Table 3.

### Characteristics associated with having no prior Hepatitis B virus infection among participants screened for the SiVET studies in Kenya and Uganda (2014–2017)

We present the results of the logistic regression analyses of having no prior HBV infection among participants who had complete data on HBsAg and HBcAb-Total (n = 1265).

In the unadjusted analysis, participants from Kampala (AOR 2.19; 95% CI 1.64–2.94) and Nairobi (AOR 2.79; 95% CI 2.06–3.81) had significantly higher odds of having no prior HBV infection than participants from the two fishing communities around Entebbe. Having a formal occupation was associated with lower odds of having no prior HBV infection than having an informal high-risk occupation (AOR 0.44; 95% CI 0.29–0.67).

In the adjusted analysis, when compared to the youngest age group (≤24 years), the odds of having no prior HBV infection were lower among those aged 25–29 years (AOR 0.51; 95%CI 0.36–0.71) and those aged ≥30 years (AOR 0.35; 95% CI 0.25–0.49). Taking participants from Entebbe with informal high-risk occupations as the reference group, participants had a higher likelihood of having no prior HBV infection if they had informal high-risk jobs and were either from Kampala (AOR 2.21; 95% CI 1.41–3.47) or Nairobi (AOR 2.61; 95% CI 1.72–4.00). Also, among Entebbe participants, there was no association between occupation and the likelihood of having no prior HBV infection (Table 4).

**Table 4:** Characteristics associated with having no prior Hepatitis B infection among participants screened for SiVET studies in Kenya and Uganda (n = 1265).

Variable	Categories	Screened Participants	No Prior HBV Infection	Unadjusted OR (95% CI)	p-value	Adjusted OR (95% CI)	p-value
		Total (N)	N (row %)				
Overall		1265	859 (67.9)				
Site*							
	Entebbe	558	318 (56.7)	Ref			
	Kampala	359	267 (74.4)	2.19 (1.64–2.94)	< 0.001	-	
	Nairobi	348	274 (78.7)	2.79 (2.06–3.81)	< 0.001	-	
Age group							
	<25	403	326 (80.9)	Ref		Ref	
	25–29	402	268 (66.7)	0.47 (0.34–0.65)	< 0.001	0.51 (0.36–0.71)	< 0.001
	≥30	460	265 (57.6)	0.32 (0.23–0.44)	< 0.001	0.35 (0.25–0.49)	< 0.001
Gender							
	Female	577	406 (70.4)	Ref		Ref	
	Male	688	453 (65.8)	0.81 (0.64–1.03)	0.086	0.91 (0.63–1.31)	0.623
Education*							
	Secondary+	654	463 (70.8)	Ref		Ref	
	Primary	465	295 (63.4)	0.72 (0.56–0.92)	0.010	0.79 (0.59–1.07)	0.123
	None	48	24 (50.0)	0.41 (0.23–0.75)	0.003	0.54 (0.29–1.02)	0.058
Main Occupation							
	Informal High Risk	972	683 (70.3)	Ref			
	Informal Low Risk	195	126 (64.6)	0.77 (0.56–1.07)	0.119	-	
	Formal	98	50 (51.0)	0.44 (0.29–0.67)	< 0.001	-	
Religion*							
	Christian	1034	694 (67.1)	Ref		Ref	
	Muslim	183	130 (71.0)	1.20 (0.86–1.71)	0.296	1.31 (0.90–1.92)	0.160
	Other	47	34 (72.3)	1.28 (0.68–2.55)	0.456	0.79 (0.35–1.90)	0.582
Site by occupation							
	Entebbe Informal High Risk	270	145 (53.7)	Ref			
	Entebbe Informal Low Risk	190	123 (64.7)	1.58 (1.08–2.32)	0.018	1.45 (0.97–2.17)	0.071
	Entebbe Formal	98	50 (51.0)	0.90 (0.56–1.43)	0.649	0.66 (0.40–1.10)	0.109
	Kampala Informal High Risk	354	264 (74.6)	2.53 (1.81–3.55)	<0.001	2.21 (1.41–3.47)	0.001
	Kampala Informal Low Risk	5	3 (60.0)	1.29 (0.21–9.93)	0.780	1.10 (0.17–8.76)	0.917
	Nairobi Informal High Risk	348	274 (78.7)	3.19 (2.25–4.55)	<0.001	2.61 (1.72–4.00)	<0.001

* N not equal to 1,265 because of missing data; OR = Odds ratio; CI = Confidence Interval; Ref = reference group

### Secondary analysis of characteristics associated with Hepatitis B infection among participants screened for the SiVET studies in Kenya and Uganda (2014–2017)

We performed a secondary analysis among 1,340 (98.1%) screened participants, with a test result for HBsAg. The analysis of HBsAg positivity showed that age group and sex were associated with living with HBV after adjustment for other demographic variables. Participants aged 25–29 years were more likely to be infected than those <25 years (AOR 2.40; 95% CI 1.07–5.89). Males were more likely than females to be living with HBV (AOR 6.34; 95% CI 1.84–39.96). While participants from Nairobi were least likely to be living with HBV in unadjusted analysis, site was no longer associated with HBV infection after adjustment for other demographic variables. Details are in “S1 Table”.

## Discussion

Overall, we found that HBV prevalence and immunity due to Hepatitis B vaccination were low among participants who were screened for SiVET studies in Uganda and Kenya with two thirds of participants having test results suggesting no prior HBV infection. We did observe significant heterogeneity among study populations, with a higher prevalence of persons living with HBV in Uganda than in Kenya. This prevalence we report is lower than that seen from recent surveys among various KPs in South Africa [11] and IDUs in Mozambique [12]. However, these surveys also reported high HIV prevalence and as part of our recruitment for the SiVET, potential participants were informed about the study requiring HIV negative participants; those who already knew their status likely opted not to report for screening and, in the Kampala cohort FSWs living with HIV were not referred for SiVET screening procedures thereby explaining the low prevalence we report. Higher HBV prevalence is often associated with HIV sero-positivity [6, 26–28] and this pre-selection may in part explain our lower observed prevalence.

A high proportion of our participants had no prior exposure to Hepatitis B with only 9 out of every 100 participants having immunity due to Hepatitis B vaccination. Despite this, they had continued sexual exposure which increases the risk of acute infection in adults and is shown to contribute 23.5% of hepatitis related deaths [29]. These results are important for HIV prevention trials: firstly, HIV vaccine trial designs in the era of PrEP are opting for layering or combination designs in which HIV vaccine trial participants are either allowed to access oral PrEP or are actively given oral PrEP during the trial [17]. The components of HIV PrEP (TDF/ FTC) are antivirals that are also active against HBV infection and might lead to a flare up of HBV infection in a few individuals with sub-optimal PrEP use during the vaccine trials [18, 19]. Sub-optimal PrEP adherence or pausing PrEP may happen for various reasons including pill burden, side effects, stigma and having reduced HIV risk [30, 31]. Secondly, HIV and HBV have similar modes of transmission therefore prevention and counselling messages for both infections are similar. Given the low levels of immunity to HBV that we found, HIV vaccine trials in our setting could be used as an opportunity to identify individuals that would benefit from HBV vaccination which might be difficult for KPs to access within the routine health services. Thirdly, HBV status remains part of trial selection criteria as seen in the HPTN084 trial that assessed efficacy of injectable cabotegravir compared to oral TDF/ FTC [16].

Older individuals (≥25 years) had a lower likelihood of having no prior HBV infection. As with HIV infection, older age has been associated with higher HBV prevalence among KPs [12] and the general population in East and West Africa [6, 28, 32], attributable to the longer periods of continued sexual exposure to HBV. Older KPs are also likely to have a higher number of life-time sexual partners, a factor that has been associated with HBV prevalence [33, 34]. We observed that older individuals (≥25 years) had lower proportions who had no prior HBV infection and the proportion who were currently living with chronic infection was 3–4 times higher when compared to younger participants <25 years. This also indicates progression of HBV infection acquired during childhood leading to chronic infection among adults. The Kampala and Entebbe SiVET participants had not benefited from childhood immunization because Hepatitis B immunization for children was only introduced in Uganda in 2002.

Overall, being from Kampala or Nairobi irrespective of main occupation was associated with a higher likelihood of having no prior HBV infection. The likelihood of having no prior HBV infection was up to 2.5 times higher among individuals whose main job was categorized as informal high risk if they were from Kampala and Nairobi compared to their counterparts in Entebbe. A similar pattern was seen among individuals from Kampala with informal low risk jobs whose likelihood of having no prior HBV infection was higher than individuals from Entebbe with informal high-risk jobs. These findings suggest that site rather than occupation is associated with the odds of having no prior HBV infection and could be explained by the different site recruitment procedures. Entebbe recruited directly from two fishing communities, Kampala recruited from the Good Health for Women Project (GHWP) clinic serving FSWs while Nairobi recruited from the Sex Workers Outreach Program (SWOP-Kenya) clinics serving FSWs and MSM. The GHWP and SWOP-Kenya clinics had been in existence since 2008 [9] and 2013 respectively and both routinely offered services including HIV risk reduction counselling, anti-retroviral therapy (ART), free condoms and family planning services, and diagnosis and treatment of sexually transmitted infections (STIs). In addition, at the time of the SiVETs, SWOP-Kenya provided HIV pre-exposure prophylaxis (PrEP), post-exposure prophylaxis (PEP), psychosocial and mental health support and adolescent health services while the GHWP clinic also screened for alcohol use disorders and offered counselling or referral depending on the level of alcohol use. HIV and HBV share transmission modes therefore, HIV risk reduction counselling and condoms provided at the clinics also reduce the risk of HBV infection. Indeed, studies done elsewhere indicate that increased condom use is associated with reduced risk of HBV infection [35, 36]. In addition, STI screening and treatment that was provided at the GHWP and SWOP clinics may further reduce the risk of HBV infection because STIs (ulcerative and non-ulcerative) have also been associated with HBV infection [37, 38]. The protective effect of site was higher for Nairobi compared to Kampala and is likely explained by the provision of PrEP at the SWOP clinics which was not yet available at the GHWP clinic at the time of SiVET study. Therefore, even though SiVET participants were all considered KPs, those recruited from already existing cohorts/ clinics that provided HIV risk reduction services were at reduced risk for HBV.

### Limitations

The results of this work are comprised of participants undergoing screening from three SiVETs, each of which had their own enrollment criteria and target populations. Because screening efforts encouraged persons without HIV to enroll, and HIV/ HBV co-infections are not uncommon, our estimates of HBV are likely underestimates, even if they might approximate what one might see when screening for an HIV vaccine trial. The results we present here are therefore likely not generalizable to the wider populations of persons not participating in clinical trials. We had missing data for some of the parameters used to ascertain HBV status due to differences in how sites screened for HBV. We therefore had 101 participants with unknown status who were not included in the logistic regression analysis. Data were also missing for some baseline variables i.e., 98 participants with known HBV status were missing education level. However, the multivariable models with and without the number of participants with missing data gave similar results. As we note above, our screening and recruitment efforts focused on persons without HIV which may account for our lower observed prevalence of HBV.

## Conclusions and recommendations

HBV prevalence and immunity due to vaccination are low among potential HIV vaccine trial participants and a high proportion have no prior exposure to HBV. Younger individuals and those recruited from existing cohorts/ clinics are more likely not to have prior HBV infection. HBV screening should continue for HIV vaccine trials layered or combined with oral PrEP and, scale up of Hepatitis B vaccination to populations at risk should be accelerated and integrated within existing HIV prevention programs. HIV prevention trials should not only screen out individuals living with HBV but should also use trials as a platform to extend HBV vaccination to KPs that need it.

## Supporting information

S1 Table Characteristics associated with Hepatitis B surface antigen positivity among participants screened for SiVET studies in Kenya and Uganda (n = 1340). (PDF)

S1 Dataset (XLSX)
