# Supplementary material for: Hepatitis B status and associated factors among participants screened for simulated HIV vaccine efficacy trials in Kenya and Uganda
Source: PLoS One. 2023 Jul 17;18(7):e0288604. doi: 10.1371/journal.pone.0288604 (PMC10351693; doi:10.1371/journal.pone.0288604)
Supplement: S1 Table — (PDF) [file pone.0288604.s001.pdf]

Supplementary Table 1: Characteristics associated with Hepatitis B Surface Antigen Positivity among Participants Screened for SiVET studies in Kenya and Uganda (n=1340)

Variable	Categories	Screened Participants Total (N)	HBsAg Positive N (row %)	Unadjusted OR (95% CI)	p-value	Adjusted OR (95% CI)	p-value
Overall		1340	47 (3.5)				
Site							
	Entebbe	633	31 (4.9)	Ref			
	Kampala	359	15 (4.2)	0.85 (0.44-1.56)	0.605	-	
	Nairobi	348	1 (0.3)	0.06 (0.00-0.26)	0.005	-	
Age group							
	<25	436	8 (1.8)	Ref		Ref	
	25- 29	418	22 (5.3)	2.97 (1.36-7.19)	0.009	2.40 (1.07-5.89)	0.041
	≥30	486	17 (3.5)	1.94 (0.85-4.80)	0.127	1.61 (0.69-4.09)	0.288
Gender							
	Female	589	17 (2.9)	Ref		Ref	
	Male	751	30 (4.0)	1.40 (0.77-2.62)	0.276	6.34 (1.84-39.96)	0.013
Education*							
	Secondary+	688	21 (3.1)	Ref		Ref	
	Primary	505	23 (4.6)	1.52 (0.83-2.97)	0.177	1.19 (0.61-2.36)	0.611
	None	49	2 (4.1)	1.35 (0.21-4.80)	0.690	0.98 (0.15-3.74)	0.976
Main Occupation							
	Informal High Risk	1020	30 (2.9)	Ref			
	Informal Low Risk	213	14 (6.6)	2.32 (1.18-4.38)	0.011	-	
	Formal	107	3 (2.8)	0.95 (0.23-2.73)	0.936	-	
Religion*							
	Christian	1092	40 (3.7)	Ref		Ref	
	Muslim	199	7 (3.5)	0.96 (0.39-2.04)	0.920	0.80 (0.32-1.74)	0.599
	Other	48	0 (0.0)	-	-	-	
Site by occupation							
	Entebbe Informal High Risk	318	14 (4.4)	Ref			
	Entebbe Informal Low Risk	208	14 (6.7)	1.57 (0.73-3.39)	0.248	1.94 (0.87-4.34)	0.104
	Entebbe Formal	107	3 (2.8)	0.63 (0.14-1.97)	0.469	0.60 (0.13-2.10)	0.463
	Kampala Informal High Risk	354	15 (4.2)	0.96 (0.45-2.04)	0.916	4.80 (1.19-32.82)	0.052
	Kampala Informal Low Risk	5	0 (0.0)	-	-	-	
	Nairobi Informal High Risk	348	1 (0.3)	0.06 (0.00-0.31)	0.008	-	

* N not equal to 1,340 because of missing data; OR= Odds ratio; CI= Confidence Interval; Ref = Reference Group
